# Telesurgery in prostate cancer: a systematic review of clinical applications and future directions

**DOI:** 10.1038/s41391-026-01090-y

**Published:** 2026-02-20

**Authors:** Boran Aksakal, Nicolas A. Soputro, Abdulrahman Al-bayati, Milagros Secin, Riccardo Autorino, Jihad Kaouk, Ruben Olivares

**Affiliations:** Glickman Urological Institute, Cleveland Clinic, Cleveland, OH USA

**Keywords:** Prostate cancer, Cancer therapy

## Abstract

**Introduction:**

Telesurgery represents an emerging frontier in the surgical management of prostate cancer, offering innovative solutions to expand access to specialized care across geographic and institutional barriers. This systematic review aims to evaluate the feasibility, clinical outcomes, and technical considerations of telesurgical applications in the treatment of prostate cancer.

**Methods:**

A comprehensive literature search was conducted across MEDLINE (via PubMed), Embase, Scopus, and Web of Science in June 2025 to identify all consecutive clinical studies published from 2001 through June 2025 that involved telesurgical approaches for the management of clinically significant, localized prostate cancer. Eligible studies were screened and reviewed according to PRISMA guidelines, and a narrative synthesis was performed.

**Results:**

A total of six studies met the inclusion criteria, demonstrating successful telesurgical procedures involving 7 patients, with six procedures pertaining to Robot-Assisted Radical Prostatectomy (RARP) and one case of High-Intensity Focused Ultrasound (HIFU) focal therapy. All procedures were completed successfully over distances ranging between 1 and 11,412 km. The most commonly utilized network infrastructure included 5 G wireless and wired fiber-optic broadband systems. With a round-trip latency ranging between 6 ms and 464 ms, no technical failures and no clinically meaningful delays perceived by the surgeons were reported.

**Conclusions:**

Herein, we demonstrated the feasibility and safety of radical and focal robotic telesurgical procedures for the management of prostate cancer. Despite its successes and benefits in democratizing patient care and surgical education, challenges surrounding its cost, regulatory frameworks, and standardization of care may continue to pose limitations, underscoring the need for further research and policy innovation.

## Introduction

Telesurgery, which enables surgeons to perform procedures remotely using robotic systems connected through high-speed networks, represents a groundbreaking advancement in surgical care. A major milestone in this field was achieved by Marescaux et al. who performed the first transatlantic cholecystectomy between New York and Strasbourg, effectively demonstrating the feasibility and potential of remote surgical intervention [1].

Prostate cancer is the most prevalent cancer in men worldwide, imposing a substantial burden on healthcare systems and patients alike [2]. Over the past decades, robotic surgery has rapidly evolved to become the gold standard for the treatment of localized prostate cancer, offering improved precision, reduced invasiveness, and better functional outcomes compared to traditional open surgery [3]. As robotic technology continued to advance, the concept of telesurgery also began to emerge, in which the surgeon operates from a remote location by controlling a robotic console. In 2008, Sterbis et al. reported the first telesurgical procedure in urology, performing a nephrectomy on a porcine model using the da Vinci system (Intuitive Surgical, Sunnyvale, CA) [4]. Subsequently, telesurgical robotic-assisted radical prostatectomy (RARP) procedures have been successfully performed on human patients across multiple regions worldwide using various robotic platforms [5–9].

Moreover, the treatment of localized prostate cancer is no longer limited to RARP. Novel, minimally invasive therapies such as High-Intensity Focused Ultrasound (HIFU) have emerged as effective alternatives, and early telesurgical applications have also begun to take shape in this field [10]. However, despite these technological advancements and the rapid integration of robotic surgery into urologic practice, telesurgery itself has not yet been adopted into routine clinical care as swiftly or extensively [9]. The slower integration likely reflects a combination of unresolved ethical concerns, the lack of a standardized international legal framework for cross-border practice, and persistent network-infrastructure constraints, particularly the need for low-latency, reliable, and redundant links that are unevenly available across regions [11]. Critical issues such as patient informed consent, data privacy, liability, and cross-border jurisdiction remain contentious and poorly defined, significantly hindering broader clinical implementation [12, 13].

This systematic review aims to comprehensively analyze the current evidence on telesurgery in the management of prostate cancer. We focus on key applications, including robotic-assisted procedures and HIFU, while evaluating clinical outcomes, technical feasibility, limitations and the future directions of this evolving field.

## Materials and methods

### Protocol and registration

This systematic review was conducted in accordance with the Preferred Reporting Items for Systematic Reviews and Meta-Analyses (PRISMA) guidelines. The protocol for this review was prospectively registered in the International Prospective Register of Systematic Reviews (PROSPERO) under the registration ID: CRD420251066163.

### Literature search

A comprehensive literature search was performed in June 2025 using MEDLINE (via PubMed), Embase, Scopus, and Web of Science to identify studies involving telesurgical techniques in the treatment of prostate cancer. Search terms included combinations of *telesurgery, remote surgery, tele-surgery, distant surgery, teleoperative, remote robotic surgery, robotic telepresence, telepresence surgery*, and *telementoring* with *prostate cancer, prostate neoplasm, prostatic neoplasms*, and *prostate*. Searches were conducted in title and abstract fields, with equivalent strategies adapted per database. Studies published from 2001 through June 2025 were screened for inclusion, and only English-language articles were retained. Reference lists of included studies and relevant reviews were also screened manually.

### Eligibility criteria

Studies were eligible if they involved adult patients undergoing prostate cancer interventions using telesurgery. Eligible designs included primary research such as randomized controlled trials, cohort studies, case series, and case reports; comparative designs were not required. We excluded studies pertaining to telementoring, those addressing benign indications or non-prostate cancer interventions, animal, cadaveric, or simulator models, and non-original reports such as editorials, narrative reviews, conference abstracts, and opinion pieces without patient-level data.

### Study selection and data extraction

All screening steps were independently performed by two reviewers (B.A. and A.A.), beginning with title and abstract screening, followed by full-text assessment of potentially eligible studies. Disagreements were resolved through discussion or, when necessary, by consultation with a third reviewer (N.S.). Data extraction was conducted independently by the same reviewers using a standardized, pilot-tested Excel form. Collected data included baseline study characteristics, procedural details, and outcome measures grouped into five domains: technical/network performance, operative outcomes, postoperative recovery, oncological outcomes, and regulatory/logistical aspects.

Technical and network performance were predefined as round-trip latency (ms), backup system, connection modality, geographic distance between sites, and the frequency of interruptions or technical failures. Operative outcomes included procedure duration, estimated blood loss, intraoperative complications, and any conversions to alternative techniques. Postoperative recovery included hospital length of stay, catheterization time, and postoperative complications graded using the Clavien–Dindo classification. Oncological outcomes included positive surgical margins (PSM) and biochemical recurrence (BCR). When reported, regulatory and logistical considerations were also recorded, including licensure and medico-legal issues, institutional credentialing, and readiness for telesurgical implementation.

### Risk of bias assessment

The methodological quality of included studies was independently assessed by two reviewers (B.A. and A.A.). Case series and case reports were appraised using the relevant checklists from the Joanna Briggs Institute (JBI). Any disagreements between reviewers were resolved through consensus following discussion.

### Data synthesis

Narrative synthesis was performed. The findings were reviewed thematically and organized to reflect variations in disease indication, technological approach, geographic or institutional context, and clinical outcomes.

## Results

### Study characteristics

**Fig. 1:**
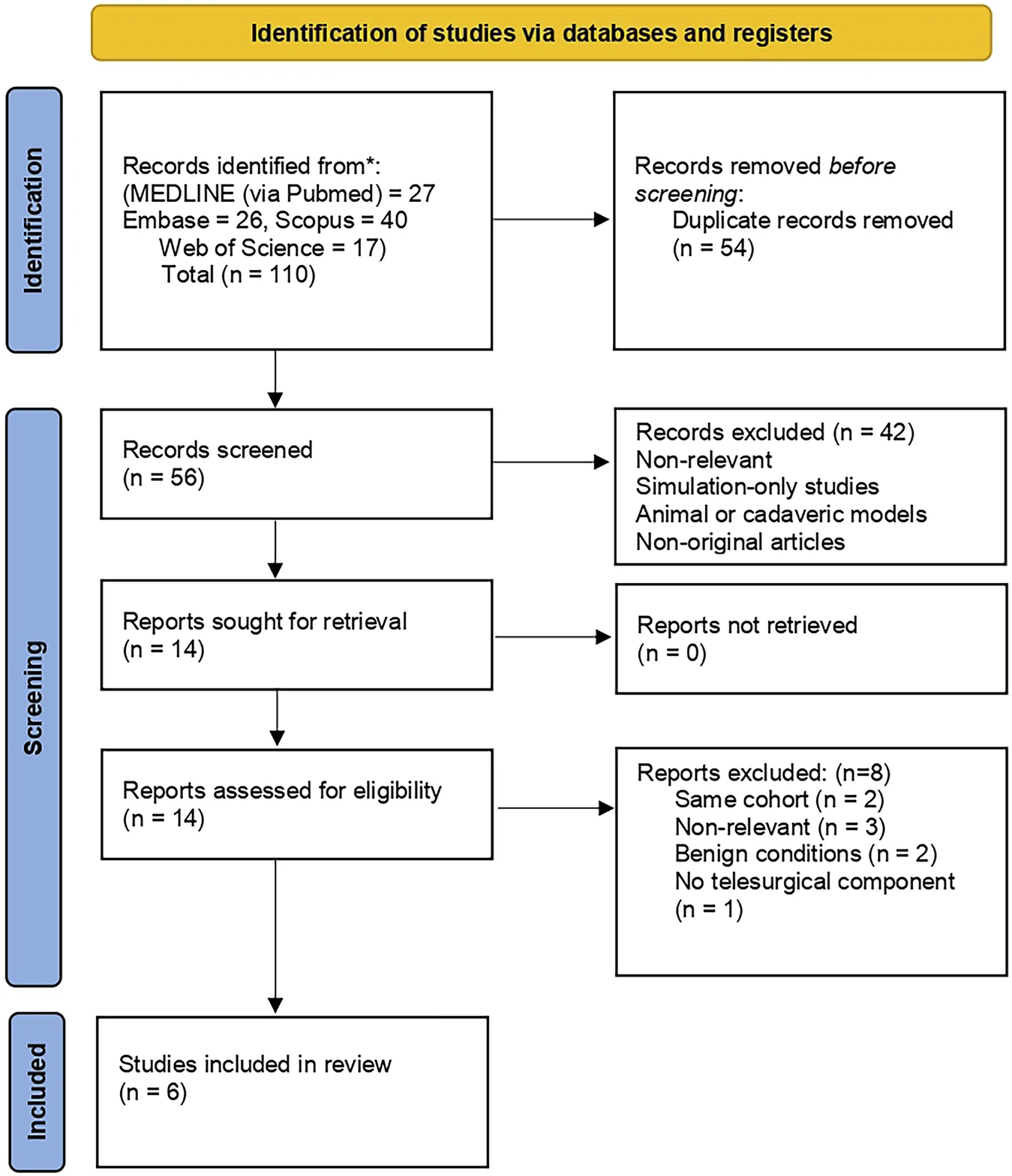
PRISMA flow diagram for study selection.

Our literature search initially yielded 110 records across four databases. After the removal of 54 duplicates, 56 unique records were screened by title and abstract. Of these, 42 were excluded for not meeting the inclusion criteria. Fourteen full-text articles were retrieved for further assessment. Following full-text review, eight studies were excluded for the following reasons: overlapping patient cohorts, irrelevance to research question, focused on benign conditions rather than prostate cancer, no inclusion of telesurgical component. Ultimately, six studies were included in the final analysis. These publications spanned a range of telesurgical applications for prostate cancer, encompassing both RARP (*n* = 5) and HIFU (*n* = 1). A PRISMA flow diagram outlining the study selection process is presented in Fig. 1. Key characteristics of the included studies, including intervention type, telecommunication method, and reported outcomes, are summarized in Table 1.

**Table 1:** Summary and technical parameters of included studies.

Author publication year	Institution	Operation	Patient’s location - surgeon console	Max distance (km)	Robotic platform	Patients (*n*)	Round-trip network latency (ms)	Type of network	Backup system
Olivares et al. 2025 [10]	Cleveland Clinic	HIFU	Abu Dhabi, UAE and Cleveland, Ohio	11,412	Focal One Robotic HIFU Device	1	Average: 115	SD-WAN	N/R
Zhou et al. 2025 [5]	Gansu Provincial Hospital, Lanzhou, China	RARP	China and China	52	TRSS	1	Average: 140 min: 31 Max: 464	Wireless 5 G network	Wired 5 G line
Aldousari et al. 2025 [6]	Department of Urology, Sabah Al-Ahmad Urology Center, Kuwait City, Kuwait	RARP	Kuwait City and Shanghai	6935	TRSS	1	Average: 181.4 min: 176 Max: 304	Wired fiber optic broadband	5 G wireless broadband
Moschovas et al. 2025 [7]	University of Central Florida - UCF, Florida, USA	RARP	Luanda and Luanda	1	TRSS	2	Average: 6 and 8	Dedicated fiber-optic	N/R
Moschovas et al. 2024 [8]	University of Central Florida - UCF, Florida, USA	RARP	Beijing and Harbin	1300	Edge Medical robotic platform MP1000	1	Median: 22 (IQR 22–22)	5 G network with OTN	OTN line
Rogers et al. 2024 [9]	AdventHealth Global Robotics Institute, Kissimmee, FL, USA	RARP (Triple Console)	Hainan, Hunan and Beijing	2600	KangDuo Endoscopic Surgical Robot	1	Max: 45	5 G and fixed networks	IPsec VPN tunnel

*RARP* Robot-Assisted Radical Prostatectomy, *HIFU* High-Intensity Focused Ultrasound, *SD-WAN* Software-Defined Wide Area Network, *TRSS* Toumai Robotic Surgical System, *OTN* Optical Transport Network, *IPsec VPN* Internet Protocol Security Virtual Private Network, *IQR* Interquartile Range, *km* Kilometers, *n* Number of patients, *N/R* Not Reported.

### RARP telesurgery for prostate cancer

RARP represents the most commonly studied telesurgical approach in prostate cancer care [5–9]. An expanding body of evidence supports its technical feasibility and clinical safety across a broad spectrum of geographic contexts, with successful applications reported over distances ranging from short-range (1 km) to ultra-long-distance procedures (6935 km) [5–9].

A representative study of a short- to mid-range telesurgical application was reported by Zhou et al. [5], who performed a RARP over a 52 km distance using 5 G connectivity. Despite challenging technical conditions, including a peak latency of 464 ms (with an average latency of 140 ms), the procedure was completed successfully with no frame loss and consistently clear visuals, avoiding potential issues such as image freezing, signal lag, or desynchronization. Although the patient experienced an extended 11-day hospital stay, this was not due to postoperative complications, as none were reported. Instead, it reflected institutional protocols implemented during the early phase of telesurgical practice. These protocols aimed to allow for enhanced monitoring of potential postoperative complications, given that this was the institution’s first experience with remote robotic surgery.

Moschovas et al. [8] demonstrated the success of mid-range telesurgery with a complication-free RARP between Beijing and Harbin (~1300 km), utilizing the Edge MP1000 platform (Shenzhen Edge Medical Co., Ltd., Shenzhen, China). The patient had Gleason 6 (ISUP 1) prostate cancer, with a PSA level of 14 ng/mL, clinical stage cT2a, a 20 mL prostate and PI-RADS 5 nodule located in the left peripheral zone at the base. The operation, which lasted only 60 min, was supported by a high-fidelity 5 G connection backed by an optical transport network (OTN). Median latency remained at 22 ms, with no packet loss or perceptible instrument lag.

The longest telesurgical RARP procedure was reported by Aldousari et al., covering a distance of 6935 km between Shanghai and Kuwait City using the Toumai Robotic Surgical System (TRSS) (Shanghai MicroPort MedBot (Group) Co., Ltd., Shanghai, China) [6]. The patient had intermediate-risk prostate adenocarcinoma characterized by a Gleason score of 7 (3 + 4) (ISUP 2), a PSA level of 6.8 ng/mL, clinical stage cT1c disease, a PI-RADS 3 lesion, and a prostate volume of 55 mL. Notably, the surgery was executed with stable audiovisual control, an average latency of 181 ms, and no adverse events. This case underscored the critical importance of a dual-network strategy, which utilized a primary fiber-optic connection complemented by a 5 G backup, in order to ensure continuous low-latency connectivity. This approach was further supported by on-site surgical standby teams and coordinated regulatory oversight to enable the safe execution of ultra-long-distance telesurgical procedures.

Complementing these experiences, Moschovas et al. reported two telesurgical RARP cases in Luanda, Angola, using the TRSS (Shanghai MicroPort MedBot Group Co., Ltd., Shanghai, China) over a dedicated fiber-optic link [7]. Two patients had PSA 8 and 39 ng/mL, Gleason 4 + 3 and 3 + 4, and prostate volumes of 45 and 30 mL were reported. The surgeon-to-patient distance was ~1 km, with mean round-trip latency of 6 and 8 ms, no frame loss, no intra- or postoperative complications, and discharge on postoperative day 2.

Equally noteworthy was the first reported triple-console telesurgical RARP, collaboratively executed by three surgeons located in Hainan, Beijing, and Hunan, China, using the KangDuo KD-SR-01 (Suzhou KangDuo Robot Co., Ltd., Suzhou, China) [9]. The patient had high-risk prostate cancer, classified as ISUP Grade Group 4 and a PSA level of 9.0 ng/mL. Spanning distances of up to 2600 km, the procedure was completed without complications, featured a total operative time of 120 min, and involved minimal blood loss (100 mL). Moreover, seamless console transitions were achieved, with peak latency reaching only 45 ms through a hybrid fixed and 5 G network setup. Consolidated summary of the technical specifications of these procedures is presented in Table 1, and perioperative outcomes are detailed in Table 2.

**Table 2:** Perioperative outcomes of included telesurgical RARP and HIFU procedures.

Author publication year	Operative time (min)	Estimated blood loss (ml)	Intra-operative complications	Post-operative complications	Length of stay (days)	Surgical margin
Olivares et al. 2025 [10]	50	0	No	No	0.17	N/A
Zhou et al. 2025 [5]	180	120	No	No	11	N/R
Aldousari et al. 2025 [6]	200	<100	No	No	2	Negative
Moschovas et al. 2025 [7] – Patient 1	80	100	No	No	2	N/R
Moschovas et al. 2025 [7] – Patient 2	85	100	No	No	2	N/R
Moschovas et al. 2024 [8]	60	100	No	No	4	Negative
Rogers et al. 2024 [9]	120	100	No	No	2	Negative

*N/A* not applicable, *N/R* not reported, *ml* Milliliters, *min* Minutes.

### HIFU telesurgery in prostate cancer

A pioneering telesurgical application for focal prostate cancer treatment was completed by Olivares et al. [10], who used the Focal One® HIFU EDAP TMS S.A., Vaulx-en-Velin, France (marketed in Austin, TX, USA) device. The procedure was conducted collaboratively between Cleveland Clinic (Ohio, USA) and Cleveland Clinic (Abu Dhabi, UAE) separated by ~11,412 km. A patient with Grade Group 2 prostate cancer and a PI-RADS 4 lesion in the left apical zone underwent successful HIFU ablation in 50 min, with a measured round-trip latency of 115 ms and no perceptible audiovisual delay. Real-time communication was maintained via Microsoft Teams, supported by a software-defined wide area network (SD-WAN) infrastructure. No intraoperative or postoperative complications were reported, blood loss was negligible, and the patient ambulated within 4 h. The technical setup of the case, including network configuration, latency, and communication platform, is detailed in Table 1. Perioperative outcomes, including procedural time, blood loss, complications, and recovery metrics, are presented separately in Table 2.

### Risk of bias assessment

All included studies were case reports (*n* = 4) or a case series (*n* = 2); therefore, risk of bias was assessed using the JBI Critical Appraisal Checklists. Case reports were judged at low risk of bias, while the case series by Zhou et al. [5] and Moschovas et al. [7] were rated moderate due to incomplete follow-up. Domain-level details are provided in Table 3.

**Table 3:** Risk of bias assessment using JBI checklists.

Study	Type	Patient demographics clearly reported	Intervention clearly described	Outcomes clearly reported	Follow-up adequate	Adverse events described	Takeaway lessons/clinical insight	Overall risk of bias
Olivares et al. 2025 [10]	Case Report	Yes	Yes	Yes	Yes	Yes	Yes	Low
Zhou et al. 2025 [5]	Case Series	Yes	Yes	Yes	Unclear	Yes	Yes	Moderate
Aldousari et al. 2025 [6]	Case Report	Yes	Yes	Yes	Yes	Yes	Yes	Low
Moschovas et al. 2025 [7]	Case Series	Yes	Yes	Yes	Unclear	Yes	Yes	Moderate
Moschovas et al. 2024 [8]	Case Report	Yes	Yes	Yes	Yes	Yes	Yes	Low
Rogers et al. 2024 [9]	Case Report	Yes	Yes	Yes	Yes	Yes	Yes	Low

Case reports and case series were appraised with the appropriate Joanna Briggs Institute (JBI) checklists. “Yes” means the criterion was met; “Unclear” indicates insufficient or incomplete reporting, often for follow-up. Overall risk of bias is a holistic judgment based on JBI domains, and study type follows the authors’ original classification.

## Discussion

Prostate cancer exceeds 1.4 million new cases annually worldwide, yet access to high-quality surgical treatment is uneven [2]. Over the past two decades, robotic surgery has become the gold standard for localized prostate cancer [3], but in many low- and middle-income settings, the scarcity of trained surgeons limits the impact of deployed systems. Telesurgery aims to bridge this expertise gap by enabling remote operation and expanding access to expert-level care [13–15].

Despite early successful initiatives [1, 4], the adoption of telesurgery in urology has not progressed as rapidly as robotic surgery overall. Its broader implementation has been hindered by unresolved regulatory, ethical, and logistical challenges that remain particularly relevant in cross-border or resource-limited contexts [16]. In addition to regulatory, ethical, and logistical barriers, cost remains one of the most significant challenges to the wider adoption of telesurgery [17].

This systematic review aims to comprehensively assess the current evidence on telesurgery in the management of prostate cancer. We highlight its feasibility and safety across various procedural platforms including RARP and HIFU while also identifying key limitations, regulatory constraints, and future considerations for wider clinical adoption.

Early telesurgical research in porcine models shaped the clinical development of remote prostate surgery. Marescaux et al.’s landmark transatlantic operation using the ZEUS system demonstrated that complex procedures can be performed safely across long distances [1]. Although not urologic, this pioneering effort provided a compelling technological proof of the telesurgery concept. Building on this foundation, Sterbis et al. performed the first urologic telesurgical procedures in porcine models using the da Vinci system [4], highlighting the field’s sensitivity to network performance. The central determinant of safe telesurgical performance is end-to-end latency, defined as the interval between a console command and the instrument’s response. Functional control is generally maintained when latency remains below the 200 ms threshold; higher delays can disrupt psychomotor coordination and visual feedback [18]. Consistent with this threshold, Sterbis et al. demonstrated in porcine models that delays approaching 900 ms caused lagging instrument response and suboptimal console visualization, underscoring the risks associated with high-latency environments [4].

With modern networks, feasibility improves. Patel et al. demonstrated that complex urologic procedures can be performed across the Pacific in porcine models using hybrid 5 G and fiber-optic links; consistently low latency enabled uninterrupted surgical flow without perceptible delay or control issues [19]. Collectively, these preclinical experiences clarified the importance of latency thresholds, infrastructure demands, and workflow design, laying a foundation for translating telesurgical RARP into human clinical practice.

Building on the experimental groundwork, early human telesurgical prostate procedures have yielded encouraging results. Across clinical telesurgical cases, average latency remained below the 200 ms threshold. Moschovas et al [8]. reported a median round-trip latency of 22 ms, whereas Aldousari et al. [6], Moschovas et al. [7], and Olivares et al [10]. reported mean round-trip latencies of 181 ms, 6–8 ms, and 115 ms, respectively, without audiovisual disruption or instrument lag. Brief spikes above 200 ms occurred in some reports [5, 6] but did not interrupt surgery, likely because the mean latency stayed within target range. These observations support that while peaks can occur, average latency may be a more meaningful indicator of telesurgical performance than isolated spikes [20]. Taken together, contemporary infrastructures, including 5 G and SD-WAN, increasingly support real-time telesurgical workflows across diverse distances.

Ensuring uncompromised operative outcomes is critical when telesurgery is used to deliver care in underserved regions where access to high-volume expertise is limited. In our review, clinical results from telesurgical prostate procedures were within expected ranges for contemporary cohorts, supporting feasibility and a favorable safety profile. Across reported telesurgical RARP cases [5–9], key perioperative parameters, including operative time, estimated blood loss, intraoperative and postoperative complication rates, length of stay, and positive margin status, were within accepted benchmarks and consistent with contemporary robotic cohorts. Where reported, procedures were completed without conversion. The single telesurgical HIFU case also showed a favorable intraoperative course and rapid recovery, with discharge at 4 h and no compromise of clinical safety or oncologic adequacy [10]. Collectively, these findings suggest that, with appropriate infrastructure and team configuration, telesurgery can reproduce expected perioperative standards while extending expert-level care across distance.

Among the reported cases, the triple-console telesurgical RARP performed by Rogers et al. represents a notable advancement in collaborative remote surgery [9]. By enabling three surgeons across different locations to participate in real time, the setup demonstrated not only technical feasibility but also educational potential. This configuration allowed for dynamic intraoperative collaboration and may support mentorship during live procedures, aligning with previous findings that multi-console systems enhance surgical training and safety [21, 22]. As telesurgery evolves, such architectures could play a critical role in promoting global expertise sharing and team-based surgical care [9].

In parallel with the evolution of surgical techniques, the robotic platforms utilized in telesurgical urologic surgery have also diversified in terms of design philosophy, console configuration, and collaborative capacity [5–9]. The Edge MP1000 (Shenzhen Edge Medical Co., Ltd., Shenzhen, China) features a closed-console setup and integrated communication architecture, supporting efficient single-surgeon telesurgical prostatectomy without visual or control delays [8]. The KD-SR-01 system offers an open-console design and supports triple-console functionality, allowing multiple surgeons to participate remotely in real time. Although initial setup and console times may be slightly longer, the system maintains clinical safety and offers a favorable learning curve [23]. In comparison, the TRSS platform (Shanghai MicroPort MedBot (Group) Co., Ltd., Shanghai, China) supports dual-console use and has demonstrated success in ultra-long-distance telesurgery, highlighting its potential for distributed collaboration and training scenarios [24]. Beyond its ultra-long-distance application, the same TRSS (Toumai) platform has also been used in a same-city configuration in Luanda, Angola, achieving single-digit latency over dedicated fiber and uneventful perioperative courses [7]. A broader overview of representative telesurgery systems and the level of supporting evidence is provided in Table 4. These considerations form an important backdrop for understanding the telesurgical cases included in this review.

**Table 4:** Representative robotic and energy-based systems relevant to telesurgery.

System (manufacturer)	Clinical status	Evidence for telesurgery	Representative use-case(s)
A) Systems represented in the included studies			
Focal One Robotic HIFU Device (EDAP TMS)	Commercial energy-based system	Human clinical telesurgery	Prostate focal therapy (HIFU)
KangDuo Endoscopic Surgical Robot (KangDuo)	Commercial robotic platform	Human clinical telesurgery	Robot-assisted radical prostatectomy (urology)
Edge Medical robotic platform MP1000 (Edge Medical Robotics)	Commercial robotic platform	Human clinical telesurgery	Robot-assisted radical prostatectomy (urology)
TRSS (Toumai Robotic Surgical System)	Commercial robotic platform	Human clinical telesurgery	Robot-assisted radical prostatectomy (urology)
B) Representative systems reported outside the included set			
ZEUS (Computer Motion) [1]	Legacy platform	Human clinical telesurgery (historical milestone)	Landmark transcontinental telesurgery (general surgery)
da Vinci (Intuitive Surgical) [4]	Commercial platform; remote use described as experimental/prototype	Pre-clinical or technical demonstrations	Remote robotic operation feasibility demonstrations

*HIFU* High-Intensity Focused Ultrasound, *TRSS* Toumai Robotic Surgical System.

Reported experience underscores that communication instability is a distinctive intraoperative risk in telesurgery. A key limitation of telesurgical RARP is the dependence on an on-site team for time-critical management of intraoperative complications. The workflow also introduces communication-related failure risks. If connectivity is lost or network conditions compromise safe teleoperation, predefined protocols should enable rapid bedside takeover and, when necessary, conversion to open surgery or discontinuation of the remote procedure [16]. In reported clinical implementations, this risk is mitigated through extensive pre-procedure system testing (including repeated phantom-model trials) and redundancy measures such as backup connectivity and a separate audiovisual feed [10]. By contrast, HIFU offers a practical advantage by eliminating tissue excision and thereby removing the risk of emergent conversion; if technical or procedural issues arise, the intervention can be safely aborted without another surgeon assuming control [25–27]. As an image-guided and minimally invasive modality, HIFU minimizes intraoperative bleeding and streamlines logistics, making it particularly well suited for remote prostate interventions [28, 29]. In the transcontinental case by Olivares et al. using the robotic Focal One system, focal HIFU for localized prostate cancer was delivered over ≈11,412 km without complications and with discharge at 4 h, illustrating procedural efficiency without compromising safety and highlighting potential scalability for focal telesurgical therapies [10, 29].

Despite these promising developments and successes, several issues must still be addressed before telesurgery can be widely adopted into routine clinical practice. The ethical use of telesurgical systems depends not only on technological capabilities but also on clear institutional responsibilities, open communication, and appropriate regulatory oversight. Patel et al. highlight that beyond secure network infrastructure, it is essential to clearly define the roles of all involved parties, including remote and on-site surgeons, technical teams, and device manufacturers, at all stages of the procedure: before, during, and after [30]. This is especially important when surgeries take place across different regions or countries, where licensure requirements, liability rules, and data protection standards may differ. Ensuring patient safety in such contexts requires mutual understanding around informed consent, control transfer protocols, and emergency planning [31, 32].

Accordingly, accountability for intraoperative adverse events should be prospectively allocated between the remote console surgeon and the on-site team, and documented within institutional credentialing and telesurgery-specific consent materials. In emergency scenarios, the authority to initiate immediate takeover, undocking, or conversion should be explicitly defined in advance, with the bedside team empowered to act without delay when patient safety thresholds are met. Such role delineation is especially critical in cross-border telesurgery, where professional liability, jurisdiction, and malpractice coverage may not align across participating sites [30–32]. However, the legal landscape for telesurgery remains underdeveloped. There is currently no unified international framework governing cross-border telesurgical practice, and informed-consent documents often insufficiently address telesurgery-specific issues such as the operator’s physical absence, potential technical failures, and data-security risks [33, 34]. As noted by Aldousari et al., coordinated action among regulators, legal experts, and clinical societies is essential to close these gaps and enable safe, scalable, and legally sound implementation [6].

Cybersecurity is a prerequisite for scalable telesurgery because real-time patient data and robotic control signals may be vulnerable to unauthorized access or tampering. Mitigation measures include operating over dedicated or logically isolated networks, end-to-end encryption (for example, IPsec), strict access control, and continuous monitoring with intrusion detection or prevention systems, alongside predefined fail-safe pathways for immediate on-site takeover if security or connectivity thresholds are breached [35]. Experimental integration of intrusion prevention devices and IPsec in a telesurgical setup has been shown to detect simulated attacks without compromising operability or image quality, while adding only minimal delay and without a meaningful increase in communication error rates [35]. Emerging approaches also propose privacy-preserving intrusion detection using federated learning, although real-world clinical validation remains limited [36].

Cost continues to represent another dominant barrier to the broader utilization of telesurgery. Although robotic surgical systems are increasingly being deployed globally through public and private investments, their integration into telesurgical programs requires substantial infrastructure [30]. This includes high-speed broadband, telecommunication redundancy, robotic platforms, and latency optimization technologies. These requirements are especially burdensome in low- and middle-income countries, where digital infrastructure and surgical workforce capacity remain limited [17, 19, 37]. Bart et al. further emphasize that concerns regarding financial sustainability and inadequate funding frameworks continue to hinder implementation in these regions [38].

Nevertheless, emerging literature suggests that telesurgery may yield long-term economic value by reducing travel for patients and providers, improving resource allocation, and centralizing training, which together could offset upfront costs [38, 39]. Continued advances in robotics, networks, and immersive tools may enable safe telesurgery outside tertiary centers, including in remote settings [8]. Realization will require coordinated efforts by industry, health systems, and regulators to establish scalable, ethical, and legally robust frameworks [6]. Ultimately, adoption depends on technical readiness, licensure harmonization, cost-effectiveness, and public trust, enabling more equitable, expert-driven surgical care [9].

This review has several limitations that should be acknowledged. First, the included evidence is derived from a small number of clinical cases, which limits the generalizability of the findings and precludes robust statistical analysis. Due to the narrative nature of this review, outcomes were synthesized qualitatively rather than through pooled or comparative data. Moreover, none of the included telesurgical prostatectomy studies were designed as direct head-to-head comparisons with conventional in-person procedures, making it difficult to assess the true magnitude of any potential differences in clinical efficacy or safety. Finally, long-term oncologic and functional outcomes remain unreported in the existing literature, thereby restricting the ability to evaluate the durability of telesurgical interventions in prostate cancer management. Future prospective studies with larger cohorts, standardized outcome measures, and longer follow-up are essential to validate these early observations and support broader implementation.

## Conclusion

This systematic review highlights the growing feasibility and safety of telesurgical approaches in the management of prostate cancer. RARP can be performed over long distances when robust networks, redundancy, and clear control-transfer protocols are in place, together with a skilled on-site team. HIFU may be amenable to telesurgical delivery in selected settings; however, the current evidence base is limited and warrants prospective evaluation. Future research should aim to address gaps in ethical governance, licensure harmonization, and cross-border regulatory alignment, which remain key barriers to wider adoption. With continued technological innovation, collaborative frameworks, and responsible clinical integration, telesurgery holds the potential to transform prostate cancer care by bridging disparities in access and improving patient outcomes.

## Data Availability

All data generated or analyzed during this study are included in this article and its supplementary information files. Additional data are available from the corresponding author upon reasonable request.
